# Splenic aneurysms: natural history and treatment techniques

**DOI:** 10.1590/1677-5449.190058

**Published:** 2019-12-04

**Authors:** Jamil Victor de Oliveira Mariúba

**Affiliations:** 1 Centro de Cuidados da Circulação e da Coluna, Sorocaba, SP, Brazil.

**Keywords:** aneurysm, splenic artery, endovascular procedures

## Abstract

True splenic artery aneurysms (SAA) are a rare, but potentially fatal, pathology. They are the third most common type of abdominal aneurysm, after aneurysms of the aorta and of the iliac artery, and account for almost the all aneurysms of visceral arteries. True aneurysms account for 60% of SAA and affect four times as many women as men, generally related to increased incidental or symptomatic findings that coincide with use of ultrasonography in pregnancy. Among pregnant patients, mortality after rupture is 65-75%, with fetal mortality exceeding 90%. There are multiple etiologies and it is believed that hormonal influences and changes in portal flow during gestation play an important role in development of SAA. This review discusses their history, epidemiology, pathophysiology, and diagnosis and current treatment techniques.

## INTRODUCTION

Arterial aneurysms occur when an artery expands locally to a diameter exceeding 50% of its expected size and they constitute an important clinical condition, causing mortality and morbidity.^1^ They are classified as fusiform or saccular, on the basis of morphology and dimensions,^1^ and as true aneurysms (when all layers of the artery wall expand) or pseudoaneurysms (when the artery expands and there is focal rupture of the wall).^2^

True splenic artery aneurysms (SAA) are a rare, but potentially fatal, pathology. This artery is considered aneurysmal when its diameter exceeds 1 cm^2^.

While rare, SAA are nevertheless the third most common type of abdominal aneurysm, after aneurysms of the aorta and the iliac artery, and account for almost all aneurysms of visceral arteries.^3^ The majority are asymptomatic and studies have shown that there is a risk of rupture when diameter exceeds 2 cm, with the possibility of life-threatening complications.^2^

This underscores the importance of continuous surveillance for SAA and opportune intervention if they reach the size limit.^2^

## HISTORY

A splenic artery aneurysm was described for the first time in 1770 by Beaussier,^4^ but surgical repair was only described for the first time almost two centuries later, by MacLeod and Maurice^5^, in 1940. Hoegler was the first to diagnose this type of injury preoperatively with X-rays, in 1920, as cited by Akbulut and Otan^1^. This also related that minimally invasive treatment techniques were only described in 1978, by Probost et al, in the case of transcatheter embolization. In 1993, Saw et al.,^6^ related the first case of laparoscopy-assisted SAA resection.

## EPIDEMIOLOGY

As use of axial imaging techniques has steadily increased, the rate of incidental detection of visceral aneurysms has also increased. However, the lack of a well-defined natural history for SAA has resulted in clinical management remaining based on historical guidelines.^7^

Splanchnic aneurysms account for 5% of intra-abdominal aneurysms.^1^ Splenic artery aneurysms are the most common type of visceral aneurysms (60%),^8^^-^ 12 followed by aneurysms of the hepatic arteries (20%), the superior mesenteric artery (5.9%), and the celiac artery (4%).^8^

In large autopsy series, the incidence of SAA varied from 0.01% to 0.2% of patients.^8^^,^ 13 ^-^ 17 Incidence increases to 10% among patients over the age of 60 and in people with portal hypertension.^8^^,^ 16 A study of 3,600 arteriograms found an incidence rate of 0.78%.^8^^,^ 12

True aneurysms account for 60% of SAA and affect four times as many women as men,^10^^-^ 12 ^,^ 17 ^,^ 18 which is potentially linked to increased incidental or symptomatic discovery that coincides with use of ultrasonography in pregnancy.^7^

One of the largest series of SAA available, covering two decades of experience, with 217 patients, revealed that 79% of affected patients were female, with a mean age of 61 years.^11^ Additional findings from the same study revealed that 95% of these aneurysms were isolated and the majority were asymptomatic. However, other authors have reported that up to 20% of SAA are multiple.^9^

The majority of true SAA emerge in the main body of the splenic artery. Around 74-87% originate in the distal third, 20-22% in the mid third, and less than 6% in the proximal third.^1^ However, the majority of mycotic aneurysms originate at the bifurcation of the splenic artery.^1^

Some authors classify aneurysms exceeding 10 cm in diameter as giant, but the majority use 5 cm as the threshold for this definition.^1^ A bibliographic study showed that giant SAA are 1.78 times more common in males, and that mean age at the time of diagnosis is 57.5 years for men and 52.7 years for women. These results suggest that SAA are diagnosed at a later age in male patients.^1^

Mortality of pregnant patients after rupture is 65-75%, with more than 90% fetal mortality. In patients with portal hypertension, mortality is greater than 50%. These elevated rates can be attributed to the asymptomatic nature of these aneurysms, to the rapid deterioration that follows rupture, and to the frequency of wrong diagnoses. Mortality among patients who are not pregnant is 25-36% in the same situation.^1^

## ANEURYSM OR PSEUDOANEURYSM

Splenic artery aneurysms can be true aneurysms or pseudoaneurysms.^17^ Both have become more common vascular findings because of increased use and precision of advanced imaging techniques such as computed tomography (CT) and ultrasonography, which have increased the frequency of diagnosis of degeneration of visceral arteries.^19^

Pseudoaneurysms are more common in men and are most often caused by acute or chronic pancreatitis and pancreatic pseudocysts.^17^^,^ 20 It has been stated that up to 10% of patients with pancreatitis will develop arterial complications because of activated pancreatic enzymes that digest the artery wall. The splenic artery is the vessel most often involved in these complications.^20^

Less common causes may be related to a concomitant increases in percutaneous and endovascular biliary interventions,^21^ peptic ulcers,^20^ mycotic infection of the artery wall,^9^ and trauma.^17^^,^ 20 Abdominal traumas are generally blunt, rather than penetrating, and more often intrasplenic, rather than restricted to the main splenic artery.^20^

In general, pseudoaneurysms are more prone to rupture than true aneurysms and some authors recommend that all pseudoaneurysms should be managed with interventions.^22^^,^ 23

Pain is the typical symptom in the clinical presentation of a splenic pseudoaneurysm, and the arterial phase of tomography with contrast will show the splenic artery or one of its intrasplenic branches contrasted and surrounded by hematoma. The best description for this finding is perhaps the term pulsating hematoma, which describes both the disease and the risk of rupture observed with all pseudoaneurysms. Since the majority of them involve risk of rupture in the absence of treatment, all should be treated, irrespective of size or manifestations.^9^

## ETIOLOGY AND HISTOLOGY

The reasons for development of SAA are not entirely clear. Several mechanisms have been proposed as playing a role in pathogenesis. Proposals made by Trimble and Hill remain valid, since they suggest that an aneurysmal dilatation is the result of two contributing factors: weakness of the artery wall and increased blood pressure.^8^

The most often reported risk factors are:

- portal hypertension^9^^,^ 11 ^,^ 17 ^,^ 18 ^,^ 24;
- arterial hypertension^17^^,^ 19 ^,^ 25;
- atherosclerosis^11^^,^ 17 ^,^ 18;
- diabetes^8^;
- cerebral aneurysm^8^;
- liver transplant^9^^,^ 17 ^,^ 18;
- medial fibroplasia^8^^,^ 9 ^,^ 18 ^,^ 24 ^,^ 25;
- alpha-1-antitrypsin deficiency^8^^,^ 9 ^,^ 25;
- smoking^17^;
- liver cirrhosis^17^^,^ 18;
- female sex^17^;
- splenomegaly^17^^,^ 18;
- collagen disease^8^^,^ 11 ^,^ 17 ^,^ 18;
- inflammatory conditions^11^^,^ 17 ^,^ 18;
- anomalous origin of the splenic artery^17^;
- infectious factors^8^^,^ 25;
- congenital anomalies affecting the arteries of the anterior intestine^18^;
- pregnancy,^18^ with multiparity^9^^,^ 24.

Histologically, there is loss of the tunica media, with disintegration of the elastic fibers,^18^^,^ 25 ^,^ 26 and loss of smooth muscle is the most common finding. Calcification, intimal hyperplasia, arterial dysplasia, fibromuscular dysplasia, and medial degeneration are all common histopathological findings.^1^ While a significant proportion of SAA do have calcifications and other pathological characteristics, as observed in atherosclerosis, these changes appear to be secondary to arterial degeneration, rather than the atherosclerosis being the principal underlying etiology.^25^ Subendothelial thickening and glycosaminoglycan build-up in the subintimal layer are also observed.^8^^,^ 26

## PREGNANCY

It is believed that hormonal influences and changes to portal flow during pregnancy play an important role in development of SAA,^9^ but the pathogenesis of the disease is unclear. It is assumed that increased levels of estrogen, progesterone, and relaxin in circulation during pregnancy increase blood flow and blood pressure in the splenic artery, weakening the artery wall, resulting in dilatation.^27^^,^ 28 Physiological changes of pregnancy, such as increased cardiac output, increased blood volume, and portal hypertension, also increase stress on the artery wall. The collateral effect of these changes is increased likelihood of formation and/or rupture of a splenic aneurysm during pregnancy.^8^

Although SAA are generally asymptomatic, pregnant women who present with pain in the upper left quadrant of the abdomen should be examined immediately to diagnose a possible SAA or other pathology.^21^ In order to mitigate the risk of maternal or fetal death after rupture, physicians must be aware that an SAA is a potential source of acute abdominal pains in pregnant women.^29^

Other aneurysms with large numbers of reports of rupture during pregnancy include those of the aorta, and cerebral, renal, coronary, and ovarian arteries.^8^

Multiparity appears to influence the incidence of SAA in women, since it is four times more common among multiparous women. The majority (two thirds) of these aneurysms rupture in the third trimester, and second-trimester ruptures are the next most common.^8^

Very often, ruptured SAA in pregnancy are diagnosed incorrectly, because they present with symptoms (nausea, vomiting, hypotension) that are similar to those of more common obstetric emergencies.^30^

## NATURAL HISTORY

The risk of rupture increases when the aneurysm has a diameter exceeding 2 cm.^9^ There is evidence that the majority of asymptomatic SAA, that are highly calcifed and smaller than 2 cm in people who are not pregnant will not expand significantly and can be effectively monitored with serial imaging exams, posing an insignificant risk of rupture.^7^^,^ 17 However, during pregnancy, aneurysms smaller than 2cm should be treated proactively.^8^

There is some evidence of an inverse relationship between quantity of calcification and aneurysm size and, consequently, risk of rupture. Pseudoaneurysms involve a significantly higher risk, with a spontaneous rupture mortality rate close to 100%.^17^

## CLINICAL STATUS

Splenic artery aneurysms are rare and insidious. Incidental diagnosis is often “presumptive”, based on circular calcifed shadows seen in the upper left quadrant of an abdominal X-ray.^31^

While many SAA are asymptomatic,^12^ they can also manifest with abdominal pains in the upper left quadrant, abdominal pulsating mass in the same area, or hypotensive shock secondary to rupture of the aneurysm (3 to 10% of cases).^2^

## RUPTURE

Some patients (2-10%) will suffer a spontaneous rupture, with mortality in the range of 10 to 40%. These cases may present with acute epigastric abdominal pains or abdominal pains in the upper left quadrant, pain in the left shoulder (Kehr’s sign), and hemodynamic instability,^17^ associated with nausea, vomiting, and sudden collapse.^8^

Aneurysms may rupture freely into the peritoneal cavity and gastrointestinal tract, causing gastrointestinal hemorrhage or erosion of neighboring structures, such as the splenic vein, causing arteriovenous fistulae.^32^ High blood flow across a fistula can cause mesenteric steal syndrome, which can provoke nontransmural ischemia of the small intestine.^33^

Rupture may be sudden, or can take place in two stages, which occurs in 20 to 25% of cases. The second type is characterized by initial containment within the lesser sac by omentum and/or blood clots, which block the foramen of Winslow. This is then followed by free rupture into the peritoneal cavity, when the pressure inside the lesser sac increases. Clinical presentation is in the form of abdominal pains, initially sudden, with an intermediate stable period, followed by sudden circulatory collapse.^8^

### Rupture during pregnancy

Rupture generally occurs during pregnancy, in 95% of cases, especially during the third trimester.^17^ It presents as severe, sudden-onset, non-traumatic hemodynamic shock.^25^ Placental abruption is one of the most common wrong diagnoses.^8^

However, there may be variations. Richardson et al. reported a case of SAA rupture during pregnancy that had a similar presentation to pulmonary embolism, with chest pain on the left side.^34^ Fender et al. reported a case in which the clinical signs of rupture were hidden by epidural anesthesia used during delivery.^35^

Abdominal sensitivity may be increased, but signs of shock are the most suggestive aspect. Abnormal cardiotocography during prenatal screening may also be observed during advanced pregnancy.^8^

Fetal disorders and mortality are generally the results of hypovolemia and shock. In cases of rupture, the objective is immediate resuscitation and stopping bleeding, generally by caesarean laparotomy. Splenectomy or pancreatectomy are generally performed in these cases, with ligature of the splenic artery.^8^

## DIAGNOSIS

Splenic artery aneurysms are discovered incidentally or, in symptomatic cases, when they rupture.^8^ Otherwise, they often remain undiscovered or are diagnosed late, because of the nonspecific symptoms or absence or clinical symptoms. Nonspecific symptoms include epigastric pain or pain in the upper left quadrant of the abdomen, nausea, vomiting, or anorexia.^17^

Mean diameter at the time of diagnosis is 2.1 cm and rarely exceeds 3 cm.^1^ A diagnostic suspicion of SAA can be raised by abdominal X-ray, ultrasonography, Doppler ultrasonography, computed tomography, magnetic resonance imaging, or arteriography of the abdominal aorta.^18^

### Imaging exams

- Abdominal X-ray

Although it is not the first-choice examination for SAA, an abdominal X-ray taken for other reasons may show a calcifed SAA, with a characteristic ring and a central area of transparency to the left of the first lumbar vertebral body.^9^^,^ 36

If these abnormal calcifications are found incidentally on radiographs, additional lateral views are needed to provide details and facilitate treatment planning. Calcification serves as a marker of the underlying disease and should not be interpreted as a sign of a stable process of long duration. For example, calcifications were observed in 90% of SAA that progressed to rupture.^9^

- Ultrasonography and Doppler ultrasonography

These are the preferred examinations during pregnancy, because they are not invasive and offer a good cost-benefit profile. In emergency cases they generally reveal the presence of free liquid in the abdomen and diagnosis is then confirmed by laparotomy.^8^

There are no contraindications against ultrasonographic procedures during pregnancy and they have widely substituted radiography as the number one method of fetal imaging. However, their utility is limited by operator dependence, in obese patients, shadows caused by intestinal gas, and arteriosclerosis. There is also a very high probability of missing small lesions because of the limited spatial resolution.^8^

Ultrasonography facilitates real-time assessment of the flow dynamics of the aneurysm, but can lead to underestimation of the true size of the lesion when color Doppler is used.^9^

- Computed tomography and magnetic resonance

Widespread use of CT has changed the clinical presentation of SAA, with increased detection of asymptomatic visceral aneurysms.^25^

In general, CT with contrast is the study of choice to identify an SAA. Typical findings include a low attenuation mass in contact with the splenic artery that has internal contrast in the arterial phase, if the aneurysm is patent,^9^ and CT is able to distinguish a tortuous vessel from an aneurysm.^8^

However, use of intravascular contrast media during pregnancy should always be avoided if possible, and should only be employed if absolutely essential and after discussion of the risks and benefits with the patient, in order to avoid any possible risk to the fetal liver.^8^

- Digital subtraction angiography

Digital subtraction angiography (DSA) is the gold standard for diagnosis of SAA. However, during pregnancy it is generally only used during a radiological intervention, such as coil embolization or endoluminal stenting.^8^

With regard to the safety of DSA, use of contrast should always be weighed up, as discussed above.^8^

On angiography, only the patent lumen of the aneurysm sac is opacified, which can lead to underestimation of the true size, and there is little information about relationships with neighboring soft tissues.^9^

## TREATMENT

There is no consensus on treatment of patients with SAA. Asymptomatic true aneurysms with dimensions exceeding 2 cm are at a high risk of rupture, and so treatment is recommended.^2^^,^ 3 ^,^ 8 ^,^ 11 ^,^ 12 ^,^ 25 ^,^ 37 The risk of rupture of pseudoaneurysms is unrelated to their dimensions and all should be treated.^1^ Symptomatic aneurysms in pregnant women or those planning a pregnancy, in patients with portal hypertension, or in liver transplantation candidates should be treated,^11^^,^ 38 irrespective of size.^17^

Regardless of the mechanism of formation, the essential issue for physicians remains clinical management and, specifically, the correct time to intervene. Treatment is necessary for SAA that are growing rapidly, symptomatic, or ruptured.^7^

Three-dimensional printed vascular models can be useful for planning. They are practical and cheap to produce.^39^

Treatment options include open surgical, laparoscopic, and percutaneous transabdominal repair; endovascular repair; and conservative treatment. Since the disease is rare, the majority of studies are retrospective, with few patients and so no Level 1 evidence is available.^2^ All of the management options have advantages and disadvantages.

- Surgical repair
  1. Open

Long-term results are excellent, but perioperative mortality is high.^2^ Still the gold standard treatment.^1^ Open surgery is widely employed for cases with rupture and hemodynamic instability.^17^

Surgery for SAA can have a mortality rate of 1 to 3%, together with a perioperative complication rate of 9 to 25%, due to splenic or pancreatic injury.^3^^,^ 11 ^,^ 26 Treatment without splenectomy is attempted whenever possible, because of the increased risk of bacterial infections over the long term.^9^^,^ 25

Classic options include ligature of the aneurysm with or without resection of the spleen and resection with revascularization.^40^ Generally, aneurysmectomy and reconstruction with preservation of the spleen (using short gastric arteries)^8^ is the option chosen for proximal SAA, while distal SAA requires aneurysmectomy with splenectomy and, sometimes, distal pancreatectomy, if the aneurysm is thoroughly adhered to the pancreas tail.^25^

2. Laparoscopic

Simple laparoscopic ligature of the artery proximal to the SAA combined with resection of the SAA with or without splenectomy has been increasingly employed. It is a simple, safe, and minimally invasive technique, with rapid recovery, reduced postoperative pain, and shorter hospital stay, compared with the open technique.^41^ Laparoscopic SAA repair is the laparoscopic intervention most often performed out of all types of aneurysm repair, because of the location of the SAA and resulting ease of access.^42^ The largest study of laparoscopic interventions described 16 patients and did not report conversions, reoperation, related problems, or deaths.^2^

Some authors recommend resection with tangential stapling for saccular aneurysms to preserve flow, but others warn that this type of laparoscopic treatment leaves part of the diseased artery and, therefore, could contribute to relapse.^41^

Ligature of the proximal and distal segments is considered the safest option for lesions in the mid third, since this is the segment that adheres to the pancreas. However, the risk of pancreatic injury during laparoscopic dissection of an SAA is more theoretical than real, because the artery runs separately from the pancreatic parenchyma and a plane between the two can be found.^41^

Despite its safety and applicability, this procedure demands experience and invasive ultrasonography. It is contraindicated in patients with hemodynamic instability or at risk of rupture. Laparoscopic excision may be the ideal treatment, particularly at the start of a pregnancy or in cases with small lesions. However, it is not appropriate for larger aneurysms with adherence to surrounding tissues.^1^

3. Transabdominal percutaneous

This is an option for cases in which transcatheter treatment is inappropriate or fails. The technique involves direct administration of coils or injection of thrombin into the lesion.^1^ Percutaneous access to the SAA is obtained with a fine needle, followed by injection of thrombin or release of coils. This should be considered a method of last resort, for use when all endovascular treatments have been attempted.^43^

- Endovascular repair

The current preference for endovascular treatment is based on the fact that these are procedures with low short term mortality and morbidity, are conducted under local anesthesia, and have rapid recovery after a short period in hospital.^7^

A systematic review with meta-analysis^2^ showed that the short-term results of endovascular treatment of SAA are better than open treatment. Nevertheless, open treatment is associated with fewer late complications and reinterventions during follow-up.^2^^,^ 25

- Conservative treatment

Conservative treatment does not involve immediate risk, but involves an increased risk of later aneurysm rupture, which can cause hemorrhage and risk of death.^2^

Changing risk factors for peripheral arterial disease through methods such as lifestyle changes, stopping smoking, platelet antiaggregants, antihypertensives, and statins has been suggested and appears logical, but the evidence confirming its efficacy is poor.^17^

Once absent or slow growth has been confirmed, serial imaging can be conducted every 1 to 2 years. The greatest growth seen in a patient under serial observation was in an 80-year-old woman whose aneurysm grew 2.4 to 3.0 cm over three and a half years.^7^

In common with other aneurysms, physicians should inform patients of the signs and symptoms of SAA rupture and of the importance of seeking immediate medical care in case of suspicion.^7^

One study has shown an inverse correlation between SAA calcification and the initial size of the aneurysm.^7^ The greater the quantity of calcification found in the SAA, the smaller it is at presentation. However, it has not been possible to correlate presence of calcification with risk of SAA rupture. There is no evidence of a protective role against aneurysm growth, nor that it is a factor in contraction of the SAA after endovascular treatment.^7^

### Endovascular treatment

The endovascular techniques most used include embolization with or without stent, exclusion with a covered stent, thrombin injection, Gelfoam injection (Pfizer Inc., Nova York, NY), administration of glue, plug placement, particle injection, and injection of polyvinyl alcohol.^44^

Irrespective of approach, the principal underlying treatment is “exclusion” of the aneurysm from circulation.^9^

### Step by step

#### Principles of endovascular treatment

Current approaches start with a remote percutaneous arterial access^9^ using a valved sheath. From here, the splenic artery is selected with a series of guides and catheters.^9^ A base catheter provides support for a smaller one (a microcatheter) that is used to select the vascular site for treatment.^9^ The choice of materials and methods will depend on the patient’s anatomy, on the shape of the splenic artery, and on the presence or absence of collateral circulation.^9^

## EMBOLIZATION

Embolization is the intentional endovascular placement of material to induce thrombosis of the vessel and is often the treatment of choice. There is not yet evidence to support superiority of either stenting or embolization.^9^

Materials used range from simple (for example, coils) or complex devices (Amplatzer Vascular Plug; St Jude Medical, St Paul, Minn), through metallic structures, particulate materials such as gelatin sponges (Gelfoam; Pharmacia Upjohn/Pfizer, Kalamazoo, Mich); to liquids, such as n-butyl cyanoacrylate (Trufill; Cordis Neurovascular, Miami Lakes, Fla) and ethylene-vinyl alcohol copolymer (Onyx; and V3 Endovascular/Covidien, Plymouth, Minn).^19^

Tortuosity of the splenic artery is often the principal limiting factor, impeding rapid advance of a guide sheath or a guide catheter to a point that is adequate for implementation.^45^

Prophylactic administration of antibiotics should not be forgotten, since it is recommended for embolizations involving the splenic artery.^30^

- Coils

Coils are the most widely-employed material and are available in a wide range of sizes and shapes, ranging from simple conical and cylindrical shapes to complex structures designed for specific applications.^9^

The technique consists of releasing coils into a vascular sac until it is excluded from the circulation or obliterated.^9^ This technique of “sac-packing” is well adapted for a saccular aneurysm with a narrow “neck”, which retains the coils in the sac and preserves flow through the splenic artery.^9^

A second technique, known as the “sandwich” method, involves afferent and efferent embolization of the splenic artery and the aneurysm. This method can be used in cases in which collateral flow could pressurize the lesion if only one segment of the vessel was occluded. Embolization of the afferent portion would be unsatisfactory because branches of the pancreatic, gastric, or distal arteries could act as retrograde collateral filling vessels, maintaining the aneurysm pressurized. The efferent artery is generally closed first, followed by the afferent artery. The “sandwich” method can also be used with complex configurations with multiple afferent or efferent vessels.^9^

Coils can be:

- “pushable”: placed by advancing them (using a “pushing” wire or an injected liquid) along a catheter, the tip of which is within the vascular lesion or site to be thrombosed;
- detachable: controlled and precise release of a coil can be achieved using a design involving a wire with a detachable connection which remains connected to the coil until a continuous current, hydrostatic pressure, or other force is applied. This “detachable” feature facilitates precise placement of coils and enables them to be recaptured or substituted until the precise site is reached.^9^

Several coils are often needed to achieve adequate occlusion of the vessel, depending on the scenario or on the size of the lesion.^9^

- Stenting

In addition to embolization, vascular exclusion techniques include use of covered and uncovered stents. These stents consist of a metallic structure with biocompatible material.^9^ The most often used stents are self-expanding or balloon-expandable.^1^

The benefit of using a covered stent is that it provides a new lumen along the splenic artery, excluding the vascular lesion.^9^ It also minimizes splenic infarction and the complications related to possible formation of abscesses with coil embolization.^1^

This technique is particularly useful in the context of an aneurysm with a wide neck. If the neck is large, embolization with conventional coils is imprudent, because of the increased risk of the coil becoming displaced, which could potentially lead to embolization of other important structures or to thrombosis of the main artery. One known disadvantage of covered stents that can limit their applications is difficulty advancing stents along small or tortuous vessels. They are therefore often reserved for proximal and more accessible sites.^19^

- Coils with stents

A combination of several treatment techniques may be needed in some cases, particularly giant SAA or patients with comorbidities,^1^ such as stent-assisted coil embolization. This is a combination of techniques used in certain anatomically challenging lesions. It combines use of a metal stent with coils. The metal stent is placed along the entire lesion to provide a scaffold, and a catheter is then placed through the mesh of the stent. The safety of coil embolization is markedly improved, because the coils are now “caged” behind the stent.^9^

- Liquid embolization

Liquid embolization is accomplished using agents that mold themselves to the aneurysm sac. A delivery catheter is used or they can be directly injected percutaneously. Cases that benefit from this technique include lesions with high blood flow and lesions in which the desired position of the embolic device is more distal than the tip of the catheter.^9^

Mold-forming agents such as n-butyl cyanoacrylate and ethylene-vinyl alcohol copolymer are used, delivered via controlled injection. Initially, the material flows, before hardening inside the lumen of the vascular lesion. Alternatively, agents such as gelatin sponges and thrombin (thrombin JMI; King Pharmaceuticals, Bristol, Tenn) can be injected to trigger the coagulation cascade and provoke thrombosis of the lesion.^9^

- Plug implantation

Another method of vascular occlusion involves a complex metallic structure, such as the vascular plug Amplatzer (St Jude Medical). This consists of a three-dimensional nitinol mesh that is advanced along a delivery catheter. Once at the desired site, the plug is deployed, unscrewing a detachable safety wire. It is designed for precise deployment using a single self-expanding occlusion device. Injectable liquid agents can also be used in conjunction.^9^

### Post-embolization follow-up

Imaging follow-up at 1-year intervals is recommended because of a 20% risk of reperfusion after successful coil embolization. If reperfusion occurs, the aneurysm sac is once more exposed to systemic pressures and may be at risk of rupture again.^9^

### Complications of embolization

The most frequent complications of transcatheter embolization are migration of coils, aneurysm rupture, intestinal infarct, fever, splenic infarction, and abscesses.^1^

The majority of endovascular procedures (80 to 90%) are technical successes, with just a small degree of splenic infarction. Collateral flow, predominantly through the short gastric arteries, maintains perfusion. However, the risk of splenic infarction increases with more distal embolizations.^9^

Ischemic pancreatitis is another potential complication, caused by occlusion of branches of the pancreatic artery, but rarely occurs if the collateral arterial supply is intact.^9^

Another complication theoretically associated with treatment of SAA is pneumococcal sepsis syndrome. There are currently no published recommendations on use of prophylactic vaccination against encapsulated organisms in the context of embolization of the splenic artery.^9^

Additionally, an aneurysm may recanalize, despite embolization having been successful. In such cases, reembolization or open abdominal surgical treatment may be preferable.^1^ There is even one case report of coil migration to the stomach.^46^

### Post-embolization syndrome

Post-embolization syndrome (PES) can manifest with fever, abdominal pains,^25^ ileum, platelet dysfunction, elevated white blood cell count,^47^ pleural effusion and, possibly, pancreatitis after infarction of the spleen, and is the most common complication after endovascular repair. It can potentially require prolonged hospitalization. Symptoms of PES are reported in up to 30% of patients.^25^ However, they do not correlate with splenic infarction. Some completely asymptomatic patients may have evidence of splenic infarction in routine postoperative imaging exams.^7^

Patients with distal SAA appear to be more at risk of PES and/or asymptomatic splenic infarction. Postoperative surveillance for splenic infarction and formation is justified. Patients with PES exhibit reduction in symptoms over time and surgical intervention is unnecessary.^7^

## FINAL COMMENTS

Splenic artery aneurysm is a rare pathology, but a potentially fatal one, with high mortality, primarily among pregnant patients after rupture. Diagnosis tends to be late and demands a high level of suspicion. Currently, endovascular treatment is preferred (except when there is hemorrhagic shock, in which cases, currently, laparotomy tends to be used). It has low short-term mortality and morbidity, is conducted under local anesthesia, and recovery is rapid after a short hospital stay. It is not yet known which endovascular technique is best, whether embolization with or without stent, exclusion with covered stents, injection of thrombin, injection of Gelfoam, administration of glue, plug deployment, injection of particles, or injection of polyvinyl alcohol, and larger prospective studies are needed to determine which technique is the most effective and safest.
