# Supplementary material for: Splenic aneurysms: natural history and treatment techniques
Source: J Vasc Bras. 2019 Dec 4;19:e20190058. doi: 10.1590/1677-5449.190058 (PMC6904962; doi:10.1590/1677-5449.190058)
Supplement: Translation

# Translation: Aneurismas de artéria esplênica: história natural e técnicas de tratamento

**Authors:** Jamil Victor de Oliveira Mariúba

**Affiliations:** 1 Centro de Cuidados da Circulação e da Coluna, Sorocaba, SP, Brasil.

**Keywords:** aneurisma, artéria esplênica, procedimentos endovasculares

## Abstract

Aneurismas da artéria esplênica (AAE) verdadeiros são uma patologia rara, mas potencialmente fatal. São o terceiro aneurisma abdominal mais comum, após aneurismas da aorta e da artéria ilíaca, e representam quase todos os aneurismas de artérias viscerais. Os aneurismas verdadeiros são responsáveis ​​por 60% dos AAEs e afetam as mulheres quatro vezes mais do que os homens, geralmente relacionados a uma descoberta incidental ou sintomática aumentada que coincide com o uso da ultrassonografia na gravidez. Em pacientes grávidas, a mortalidade, após a ruptura, é de 65-75%, com mais de 90% de mortalidade fetal. Têm múltiplas etiologias, e acredita-se que as influências hormonais e as alterações do fluxo portal durante a gestação desempenhem um papel importante no desenvolvimento do AAE. Esta revisão discorrerá sobre sua história, epidemiologia, fisiopatologia, diagnóstico, e as técnicas atuais de tratamento.

## INTRODUÇÃO

Os aneurismas arteriais, que ocorrem quando uma artéria apresenta um aumento focal de mais de 50% do diâmetro esperado, constituem uma importante condição clínica e resultam em mortalidade e morbidade^1^. Eles são classificados em fusiformes ou saculares, com base em sua morfologia e dimensão^1^, e em verdadeiros (como expansões de todas as camadas da parede) e pseudoaneurismas (como expansões da artéria com ruptura focal da parede)^2^.

Aneurismas da artéria esplênica (AAEs) verdadeiros são uma patologia rara, mas potencialmente fatal. Essa artéria é considerada aneurismática quando seu diâmetro é maior que 1 cm^2^.

Embora raros, AAEs são o terceiro aneurisma abdominal mais comum, após os aneurismas da aorta e da artéria ilíaca, e representam quase todos os aneurismas de artérias viscerais^3^. A maioria é assintomática, e os estudos mostraram risco de ruptura quando o diâmetro excede 2 cm, o que pode resultar em complicações ameaçadoras à vida^2^.

Isso enfatiza a importância da vigilância contínua do AAE e da intervenção oportuna, caso ele atinja o tamanho limite^2^.

## HISTÓRICO

O AAE foi descrito pela primeira vez em 1770 por Beaussier^4^, mas seu reparo cirúrgico foi descrito pela primeira vez somente quase dois séculos depois, por MacLeod e Maurice, em 1940^5^. Hoegler foi quem primeiro diagnosticou essas lesões no pré-operatório com exames radiológicos, em 1920, conforme citado por Akbulut e Otan^1^. Este também relatou que as técnicas minimamente invasivas de tratamento só foram descritas em 1978, por Probost et al., no caso da embolização transcateter. Em 1993, Saw et al.^6^ relataram o primeiro caso de ressecção de AAE assistida por laparoscopia.

## EPIDEMIOLOGIA

Com o uso cada vez maior de técnicas de imagem axial, a detecção incidental de aneurismas viscerais também aumentou. No entanto, a ausência de uma história natural bem definida para o AAE fez com que o manejo clínico fosse baseado em diretrizes históricas^7^.

Os aneurismas esplâncnicos constituem 5% dos aneurismas intra-abdominais^1^. Os AAEs são os mais comuns entre os aneurismas viscerais (60%)^8^^-^ 12, seguidos pelos aneurismas das artérias hepática (20%), mesentérica superior (5,9%) e celíaca (4%)^8^.

A incidência de AAE, em grandes séries de autópsias, variou de 0,01% a 0,2% dos pacientes^8^^,^ 13 ^-^ 17. A incidência aumenta para 10% em pacientes com mais de 60 anos e naqueles com hipertensão portal^8^^,^ 16. Em um estudo de 3.600 arteriogramas, a incidência foi de 0,78%^8^^,^ 12.

Os aneurismas verdadeiros são responsáveis ​​por 60% dos AAEs e afetam as mulheres quatro vezes mais do que os homens^10^^-^ 12 ^,^ 17 ^,^ 18, situação potencialmente relacionada a uma descoberta incidental ou sintomática aumentada que coincide com o uso da ultrassonografia na gravidez^7^.

Uma das maiores séries de AAEs disponíveis, abrangendo duas décadas de experiência, com 217 pacientes, revelou que 79% dos pacientes afetados eram do sexo feminino, com idade média de 61 anos^11^. Achados adicionais desse estudo revelaram que 95% dos aneurismas eram solitários e a maioria era assintomática; entretanto, outros autores relataram que até 20% dos AAEs são múltiplos^9^.

A maioria dos AAEs verdadeiros surge no corpo principal da artéria esplênica. Cerca de 74-87% se originam no terço distal, 20-22% no terço médio, e menos de 6% no terço proximal^1^. Por outro lado, a maioria dos aneurismas micóticos surge na bifurcação da artéria esplênica^1^.

Aneurismas maiores que 10 cm em diâmetro são classificadas como lesões gigantes por alguns autores, mas a maioria utiliza 5 cm como limiar^1^. Uma pesquisa bibliográfica revelou que AAEs gigantes são 1,78 vezes mais frequentes no sexo masculino, e a média de idade, no momento do diagnóstico, é de 57,5 ​​anos para homens e 52,7 anos para as mulheres. Esses resultados sugerem que os AAEs são diagnosticados em uma idade mais avançada em pacientes masculinos^1^.

Em pacientes grávidas, a mortalidade, após a ruptura, é de 65-75%, com mais de 90% de mortalidade fetal. Em pacientes com hipertensão portal, a mortalidade é maior que 50%. Essas altas taxas podem ser atribuídas à natureza assintomática do aneurisma, à rápida deterioração após a ruptura e a frequentes erros de diagnóstico. A mortalidade, em pacientes não grávidas na mesma situação, é de 25-36%^1^.

## ANEURISMA OU PSEUDOANEURISMA

Os AAEs podem ser verdadeiros ou falsos (pseudoaneurisma)^17^. Ambos se tornaram um achado vascular mais frequente devido ao uso e à precisão de técnicas avançadas de imagem, como a tomografia computadorizada (TC) e a ultrassonografia, que aumentaram a frequência do diagnóstico de degeneração das artérias viscerais^19^.

Pseudoaneurismas ocorrem com mais frequência em homens e são mais comumente decorrentes de pancreatite aguda ou crônica e de pseudocisto pancreático^17^^,^ 20. Afirma-se que até 10% dos pacientes com pancreatite desenvolvem complicações arteriais como resultado de enzimas pancreáticas ativadas que digerem a parede arterial. A artéria esplênica é a mais comumente envolvida nessas complicações^20^.

Causas menos comuns podem estar relacionadas ao aumento concomitante de intervenções biliares percutâneas e endovasculares^21^, úlceras pépticas^20^, infecção micótica da parede da artéria^9^ e trauma^17^^,^ 20. O trauma abdominal é geralmente contuso em vez de penetrante e, mais frequentemente, intraesplênico em vez de restrito à artéria esplênica principal^20^.

Em geral, os pseudoaneurismas são mais propensos a se romperem do que os verdadeiros, e alguns autores propuseram o manejo de todos os pseudoaneurismas com intervenção^22^^,^ 23.

A dor é o sintoma típico do quadro clínico de um pseudoaneurisma esplênico, e a tomografia com contraste demonstra, na fase arterial, a contrastação da artéria esplênica ou de um de seus ramos intraesplênicos cercados por um hematoma. Talvez o melhor descritor para esse achado seja o termo hematoma pulsátil, que descreve tanto a doença como o risco de ruptura observado com qualquer pseudoaneurisma. Como na maioria destes, existe risco de ruptura se não houver tratamento, e todos devem ser tratados, independentemente do tamanho ou da manifestação^9^.

## ETIOLOGIA E HISTOLOGIA

A razão para o desenvolvimento do AAE não é totalmente conhecida. Diferentes mecanismos têm sido propostos como tendo papel na patogênese. As propostas de Trimble & Hill permanecem válidas, quando sugeriram que a dilatação aneurismática resulta de dois fatores contribuintes: fraqueza da parede arterial e aumento da pressão arterial^8^.

As condições de risco mais relatadas são:

- hipertensão portal^9^^,^ 11 ^,^ 17 ^,^ 18 ^,^ 24;
- hipertensão arterial^17^^,^ 19 ^,^ 25;
- aterosclerose^11^^,^ 17 ^,^ 18;
- diabetes^8^;
- aneurisma cerebral^8^;
- transplante de fígado^9^^,^ 17 ^,^ 18;
- fibroplasia medial^8^^,^ 9 ^,^ 18 ^,^ 24 ^,^ 25;
- deficiência de alfa-1-antitripsina^8^^,^ 9 ^,^ 25;
- tabagismo^17^;
- cirrose hepática^17^^,^ 18;
- sexo feminino^17^;
- esplenomegalia^17^^,^ 18;
- doença do colágeno^8^^,^ 11 ^,^ 17 ^,^ 18;
- condições inflamatórias^11^^,^ 17 ^,^ 18;
- origem anômala da artéria esplênica^17^;
- fatores infecciosos^8^^,^ 25;
- anomalias congênitas que afetam as artérias do intestino anterior^18^;
- gravidez^18^, com multiparidade^9^^,^ 24.

Histologicamente, há a perda da camada média, com desintegração das fibras elásticas^18^^,^ 25 ^,^ 26, e a perda do músculo liso é o achado mais frequente. Calcificação, hiperplasia intimal, displasia arterial, displasia fibromuscular e degeneração medial são achados histopatológicos comuns^1^. Embora um número significativo de AAEs apresente calcificações e outras características patológicas, como observado na aterosclerose, essas alterações parecem ser secundárias à degeneração arterial, em vez de a aterosclerose ser a principal etiologia subjacente^25^. Notam-se também espessamento subendotelial e acúmulo de glicosaminoglicanos na camada subintimal^8^^,^ 26.

## A GRAVIDEZ

Acredita-se que influências hormonais e alterações do fluxo portal durante a gestação desempenhem um papel importante no desenvolvimento do AAE^9^. A patogênese da doença não é bem compreendida. Supõe-se que, na gravidez, um aumento nos níveis circulantes de estrogênio, progesterona e relaxina aumente o fluxo sanguíneo e a pressão sanguínea dentro da artéria esplênica, levando ao enfraquecimento da parede arterial, o que resulta em dilatação^27^^,^ 28. Alterações fisiológicas da gravidez, como aumento do débito cardíaco, aumento do volume sanguíneo e hipertensão portal, também aumentam o estresse na parede arterial. O efeito colateral dessas alterações é o aumento da chance de formação e/ou ruptura de aneurisma esplênico na gravidez^8^.

Embora os AAEs sejam geralmente assintomáticos, as gestantes que apresentem dor no quadrante superior esquerdo do abdome devem ser examinadas imediatamente para diagnosticar um possível AAE ou outra patologia^21^. A fim de mitigar o risco de morte materna ou fetal após a ruptura, os médicos devem permanecer cientes de que o AAE é fonte potencial de dor abdominal aguda em mulheres grávidas^29^.

Outros aneurismas com grande número de relatos de rompimento durante a gravidez incluem os de aorta, cerebrais, renais, coronarianos e ovarianos^8^.

A multiparidade parece influenciar a incidência de AAE em mulheres, sendo quatro vezes mais comum em multíparas. A maioria (dois terços) dos aneurismas se rompem no terceiro trimestre, seguido de rupturas no segundo trimestre^8^.

Muitas vezes, há erro diagnóstico na ruptura de AAE na gravidez, pois essa se apresenta com sintomas (náuseas, vômitos, hipotensão) semelhantes aos das emergências obstétricas mais comuns^30^.

## HISTÓRIA NATURAL

O risco de ruptura aumenta quando o aneurisma mede mais de 2 cm de diâmetro^9^. Há evidência de que a maioria dos AAEs assintomáticos, com menos de 2 cm e altamente calcificados em não grávidas não crescerão significativamente, podem ser monitorados de forma eficaz com imagens seriadas e apresentam um risco insignificante de ruptura^7^^,^ 17. No entanto, durante a gravidez, o tratamento proativo de aneurismas menores que 2 cm deve ser realizado^8^.

Existem algumas evidências de uma relação inversa entre a quantidade de calcificação e o tamanho do aneurisma e, portanto, o risco de ruptura. Pseudoaneurismas têm um risco significativamente maior, com uma taxa de mortalidade por ruptura espontânea de quase 100%^17^.

## QUADRO CLÍNICO

Os AAEs são relativamente raros e insidiosos. O diagnóstico incidental é frequentemente “presuntivo”, baseado em sombras calcificadas circulares vistas no quadrante superior esquerdo de uma radiografia abdominal^31^.

Embora muitos AAEs sejam assintomáticos^12^, pode manifestar-se como dor abdominal no quadrante superior esquerdo, massa abdominal pulsátil nesse local, ou choque hipotensivo secundário à ruptura do aneurisma (3% a 10% dos casos)^2^.

## RUPTURA

Alguns (2-10%) dos pacientes apresentarão ruptura espontânea, com mortalidade de 10-40%. Esses podem apresentar dor abdominal aguda epigástrica ou no quadrante superior esquerdo, dor no ombro esquerdo (sinal de Kehr) e instabilidade hemodinâmica^17^, associados a náuseas, vômitos e colapso súbito^8^.

Os aneurismas podem se romper livremente na cavidade peritoneal e no trato gastrointestinal, causando hemorragia gastrointestinal ou erosão em estruturas vizinhas, como a veia esplênica, resultando em fístula arteriovenosa^32^. Um alto fluxo sanguíneo através desta pode levar à síndrome do roubo mesentérico, o que pode resultar em isquemia do intestino delgado não transmural^33^.

A ruptura pode ser súbita ou acontecer em dois estágios, o que ocorre em 20 a 25% dos casos. Esta última é caracterizada pela contenção inicial dentro da retrocavidade por omento e/ou coágulos sanguíneos, que bloqueiam o forame de Winslow. Isso é seguido pela ruptura livre para a cavidade peritoneal, quando a tensão dentro da retrocavidade aumenta. A apresentação clínica é na forma de dor abdominal inicialmente súbita, com um período de tempo estável intermediário, seguido de colapso circulatório súbito^8^.

### Ruptura na gravidez

A ruptura geralmente ocorre na gravidez, em 95% das vezes, especialmente no terceiro trimestre^17^. Apresenta-se como choque hemodinâmico grave de início súbito, não traumático^25^. O descolamento de placenta é um dos diagnósticos errôneos mais comuns^8^.

Mas pode haver variações. Richardson et al.^34^ relataram um caso de ruptura de AAE na gravidez que apresentava quadro semelhante ao da embolia pulmonar, com dor torácica no lado esquerdo. Fender et al.^35^ relataram um caso em que os sinais clínicos da ruptura foram mascarados por anestesia epidural usada durante o parto.

A sensibilidade abdominal pode estar aumentada, embora os sinais de choque sejam os aspectos mais sugestivos. O rastreamento com cardiotocograma anormal também é observado em casos de gravidez avançada^8^.

O distúrbio fetal e a mortalidade geralmente são resultados da hipovolemia e do choque. Em casos de ruptura, o objetivo é a reanimação imediata e a cessação do sangramento, geralmente por laparotomia cesariana. A esplenectomia ou a pancreatectomia geralmente são empregadas nesses casos, com ligadura da artéria esplênica^8^.

## DIAGNÓSTICO

Os AAEs são descobertos incidentalmente ou, em casos sintomáticos, quando se rompem^8^. Além disso, são frequentemente ignorados ou têm um diagnóstico tardio, devido a sintomas inespecíficos ou à falta de sintomas clínicos. Os sintomas inespecíficos incluem dor epigástrica ou no quadrante superior esquerdo, náuseas, vômitos ou anorexia^17^.

No momento do diagnóstico, o diâmetro médio é de 2,1 cm e raramente ultrapassa 3 cm^1^. A suspeita diagnóstica de AAE pode ser feita por radiografia abdominal, ultrassonografia, ultrassonografia com Doppler, tomografia computadorizada, ressonância nuclear magnética e arteriografia da aorta abdominal^18^.

### Exames complementares

- Radiografia abdominal

Embora não seja o primeiro exame para AAE, a radiografia abdominal realizada por outro motivo pode revelar AAE calcificado, com um anel característico e uma área central de transparência à esquerda do primeiro corpo vertebral lombar^9^^,^ 36.

Se essas calcificações anormais forem descobertas, incidentalmente, na radiografia, imagens de corte transversais adicionais são necessárias para caracterizar e facilitar o planejamento. A calcificação serve como um marcador da doença subjacente e não deve ser interpretada como sinal de um processo estável e de longa duração. Por exemplo, calcificações foram observadas em 90% dos AAEs que progrediram para a ruptura^9^.

- Ultrassonografia e ultrassonografia com Doppler

São exames preferíveis na gravidez, porque não são invasivos e têm bom custo-benefício. Em casos de emergência, geralmente revelam a presença de líquido livre no abdome, e o diagnóstico é confirmado em laparotomia^8^.

Não há contraindicações para os procedimentos ultrassonográficos durante a gravidez, os quais substituem amplamente a radiografia como o principal método de imagem fetal. No entanto, sua utilidade é limitada pela dependência do operador, em pacientes obesos, sombra de gases intestinais e arteriosclerose. A probabilidade de perder lesões menores também é bastante alta, devido à limitada resolução espacial^8^.

A ultrassonografia facilita a avaliação em tempo real da dinâmica do fluxo do aneurisma, mas pode levar a uma subestimação do tamanho real da lesão quando o Doppler colorido é usado^9^.

- Tomografia computadorizada e ressonância magnética

O uso difundido da TC modificou a apresentação clínica dos AAEs devido ao aumento da detecção de aneurismas viscerais assintomáticos^25^.

A TC com contraste, geralmente, é o estudo de escolha para identificar um AAE. Os achados típicos incluem uma massa de atenuação baixa contínua com a artéria esplênica e que demonstra realce interno da fase arterial, se o aneurisma estiver patente^9^, e o TC consegue distinguir o vaso tortuoso do aneurisma^8^.

Mas se deve, sempre, tentar evitar o uso de meio de contraste intravascular durante a gestação, utilizando-o apenas se for absolutamente essencial e somente após discussão dos riscos e benefícios com a paciente, a fim de tentar evitar qualquer possível risco para o fígado fetal^8^.

- Angiografia por subtração digital

A angiografia por subtração digital (ASD) é o padrão-ouro para o diagnóstico de AAE. Mas na gestação, geralmente, é empregada apenas durante uma intervenção radiológica, como embolização com mola ou implante de stent endoluminal^8^.

No que diz respeito à segurança da ASD, deve-se ponderar o uso do contraste, conforme discutido acima^8^.

Na angiografia, apenas o lúmen patente do saco aneurismático é opacificado, o que pode causar subestimação do verdadeiro tamanho, e fornece pouca informação sobre as relações de tecido mole vizinhas^9^.

## TRATAMENTO

Não existe consenso sobre o tratamento dos pacientes com AAE. Os verdadeiros assintomáticos com dimensões maiores que 2 cm têm alto risco de ruptura, e portanto o tratamento é recomendado^2^^,^ 3 ^,^ 8 ^,^ 11 ^,^ 12 ^,^ 25 ^,^ 37. O risco de ruptura de pseudoaneurismas não está relacionado com as suas dimensões, e todos devem ser tratados^1^. Os aneurismas sintomáticos, em gestantes ou que planejam engravidar, em portadores de hipertensão portal, ou em candidatos a transplante hepático, devem ser tratados^11^^,^ 38, independentemente do tamanho^17^.

Qualquer que seja o mecanismo de formação, a questão essencial para os médicos continua a ser o manejo clínico e, especificamente, o momento de intervir. AAEs com aumento rápido, sintomáticos ou com ruptura requerem tratamento^7^.

Modelos vasculares impressos em três dimensões podem ser úteis no planejamento. Estes podem ser produzidos praticamente com baixo custo^39^.

Como opções de tratamento, temos os reparos cirúrgicos aberto, laparoscópico e transabdominal percutâneo; o reparo endovascular; e o tratamento conservador. Como essa doença é rara, a maioria dos estudos é retrospectiva, com poucos pacientes e, portanto, nenhuma evidência de Nível 1 está disponível^2^. Todas as opções de gerenciamento têm vantagens e desvantagens.

- Reparo cirúrgico
  1. Aberto

Demonstrou excelentes resultados a longo prazo, mas com alta mortalidade perioperatória^2^. Ainda continua sendo o padrão-ouro para o tratamento^1^. Na ruptura com instabilidade hemodinâmica, a cirurgia aberta é largamente utilizada^17^.

A cirurgia para AAE pode levar a uma taxa de mortalidade de 1% a 3%, juntamente com uma taxa de complicações perioperatória de 9% a 25%, por lesões esplênicas ou pancreáticas^3^^,^ 11 ^,^ 26. Tenta-se sempre evitar a esplenectomia, devido ao aumento do risco de infecções bacterianas a longo prazo^9^^,^ 25.

As opções clássicas incluem a ligadura do aneurisma com ou sem ressecção do baço e a ressecção com revascularização^40^. Geralmente, a aneurismectomia e a reconstrução com preservação esplênica (através das artérias gástricas curtas)^8^ são a opção para o AAE proximal, enquanto o AAE distal requer aneurismectomia com esplenectomia e, às vezes, pancreatectomia distal, se o aneurisma estiver muito aderente à cauda do pâncreas^25^.

2. Laparoscópico

A ligadura laparoscópica simples da artéria proximal ao AAE associada a ressecção do AAE com ou sem esplenectomia tem sido realizada com frequência crescente. É uma técnica simples, segura e minimamente invasiva, com rápida recuperação, diminuição da dor pós-operatória e menor permanência hospitalar, em comparação com a técnica aberta^41^. O reparo laparoscópico do AAE é a intervenção laparoscópica mais realizada de todos os aneurismas, devido à localização do AAE e seu fácil acesso^42^. O maior estudo de intervenções laparoscópicas, consistindo em 16 pacientes, não relatou conversões, necessidade de reoperação, afecções relacionadas ou mortes^2^.

Alguns autores defendem a ressecção com grampeador tangencial nos aneurismas saculares para preservar o fluxo, mas outros temem que esse tipo de tratamento laparoscópico deixe parte da artéria doente, e, portanto, possa contribuir para a recidiva^41^.

A ligadura do segmento proximal e distal é considerada mais segura nas lesões do terço médio, pois é uma região aderida ao pâncreas. No entanto, o risco de lesão pancreática durante a dissecção laparoscópica de um AAE é mais teórico do que real, porque a artéria corre separada do parênquima pancreático e um plano pode ser encontrado entre os dois^41^.

Apesar de sua segurança e aplicabilidade, esse procedimento requer experiência e ultrassonografia invasiva. É contraindicado em pacientes com instabilidade hemodinâmica ou em risco de ruptura. A excisão laparoscópica pode ser o tratamento ideal, particularmente no início da gravidez e no caso de pequenas lesões. No entanto, não é adequado para aneurismas e lesões maiores com aderências aos tecidos circundantes^1^.

3. Transabdominal percutâneo

É uma opção para casos em que o tratamento transcateter não é adequado ou falhou. Essa técnica envolve aplicação direta de mola ou injeção de trombina na lesão^1^. Realiza-se o acesso percutâneo ao AAE com agulha fina, seguido da injeção de trombina com ou sem colocação de molas. Só deve ser considerado um método de último recurso, se todos os tratamentos endovasculares tiverem sido tentados^43^.

- Reparo endovascular

A preferência atual pela terapia endovascular baseia-se no fato de que se trata de um procedimento de baixa mortalidade e morbidade no curto prazo, realizado sob anestesia local e permitindo rápida recuperação após um curto período de internação hospitalar^7^.

Em uma revisão sistemática com meta-análise^2^, mostrou-se que o tratamento endovascular dos AAEs tem melhores resultados a curto prazo do que o tratamento aberto. No entanto, o aberto está associado a menos complicações tardias e reintervenções durante o acompanhamento^2^^,^ 25.

- Tratamento conservador

O tratamento conservador não tem risco imediato, mas acarreta um aumento posterior do risco de ruptura do aneurisma, o que pode resultar em hemorragia e risco de morte^2^.

A modificação dos fatores de risco para doença arterial periférica, como mudanças de estilo de vida, cessação do tabagismo, antiagregantes plaquetários, anti-hipertensivos e estatinas já foi oferecida e parece lógica, mas as evidências que comprovem sua eficácia são ruins^17^.

Uma vez confirmada a ausência ou o crescimento lento, a imagem seriada pode ser realizada a cada 1 a 2 anos. A maior quantidade de crescimento para um paciente sob observação seriada foi o de uma mulher de 80 anos, cujo aneurisma cresceu de 2,4 a 3,0 cm ao longo de 3 anos e meio^7^.

Da mesma forma que outros aneurismas, os médicos devem informar aos pacientes os sinais e sintomas de ruptura de AAE, e a importância de procurar atendimento médico imediato em caso de suspeita^7^.

Um estudo mostrou a correlação inversa entre a calcificação do AAE e o tamanho inicial do aneurisma^7^. Quanto maior a quantidade de calcificação presente no AAE, menor ele é na apresentação. No entanto, não foi possível correlacionar a presença de calcificação com o risco de ruptura do AAE. Não há evidência de um papel protetor contra o crescimento do aneurisma, nem como um fator na contração do AAE após terapia endovascular^7^.

### Tratamento endovascular

As técnicas endovasculares mais utilizadas incluem embolização com ou sem stent, exclusão com stent recoberto, injeção de trombina, injeção de Gelfoam (Pfizer Inc., Nova York, NY), colocação de cola, colocação de plugue, injeção de partícula e injeção de álcool polivinílico^44^.

Independentemente da abordagem, o princípio do tratamento é a “exclusão” da lesão da circulação^9^.

### Passo a passo

#### Princípios do tratamento endovascular

A abordagem atual começa com acesso percutâneo arterial remoto^9^ através de uma bainha valvulada. Desse local, a artéria esplênica é selecionada com uma série de guias e cateteres^9^. Um cateter de base fornece suporte para um menor (microcateter), que é usado para seletivar a localização vascular para tratamento^9^. A escolha do material ou do método dependerá da anatomia do paciente, da conformação da artéria esplênica e da presença ou ausência de circulação colateral^9^.

## EMBOLIZAÇÃO

A embolização é a colocação endovascular intencional de material para trombose do vaso, e este é frequentemente o tratamento de escolha. Atualmente, não há evidências para apoiar a superioridade do implante de stent ou da embolização^9^.

Incluem materiais simples (por exemplo, molas) e complexos (Amplatzer Vascular Plug; St Jude Medical, St Paul, Minn), estruturas metálicas, materiais particulados, como esponja de gelatina (Gelfoam; Pharmacia Upjohn/Pfizer, Kalamazoo, Mich); e líquidos, como o cianoacrilato de n-butilo (Trufill; Cordis Neurovascular, Miami Lakes, Fla) e o etileno copolímero de álcool vinílico (Onyx; eV3 Endovascular/Covidien, Plymouth, Minn)^19^.

A tortuosidade da artéria esplênica é frequentemente o principal fator limitante que impede o avanço rápido de uma bainha guia ou de um cateter-guia para um nível adequado de implantação^45^.

Deve-se lembrar da administração profilática de antibióticos, que é recomendada para a embolização envolvendo a artéria esplênica.^30^.

- Molas

As molas são o material mais utilizado e estão disponíveis em uma ampla gama de tamanhos e formas, a partir de formas simples, cônicas e cilíndricas até estruturas complexas projetadas para uma aplicação específica^9^.

Consiste em colocar molas em um saco vascular até que esteja excluído da circulação ou obliterado^9^. Esta técnica de *sac-packing* é bem adequada para um aneurisma sacular com um “pescoço” estreito, o que permite a retenção das molas no saco e preserva o fluxo da artéria esplênica^9^.

A segunda técnica, conhecida como a técnica “sanduíche”, envolve embolização da artéria esplênica aferente e eferente ao aneurisma. Esse método pode ser usado nos casos em que o fluxo colateral possa pressurizar a lesão se apenas um segmento do vaso estiver ocluído. A embolização da porção aferente seria insatisfatória, uma vez que ramos das artérias pancreáticas, gástricas ou distais, atuando como vasos colaterais de preenchimento retrógrado, podem manter o aneurisma pressurizado. A artéria eferente é geralmente fechada primeiro, seguida pela artéria aferente. A técnica “sanduíche” também pode ser aplicada em configurações complexas de múltiplos aferentes ou eferentes^9^.

As molas podem ser:

- “empurráveis”: colocadas por avançamento (com um fio “empurrador” ou um líquido injetado), por meio de um cateter cuja ponta está dentro da lesão vascular ou local a ser trombosado;
- destacáveis: uma implantação controlada e precisa de uma mola pode ser alcançada com um *design* que envolve um fio de conexão destacável, que permanece ligado à mola até que uma corrente contínua, pressão hidrostática ou outra força seja aplicada. Esse recurso “destacável” facilita a implantação precisa das molas e permite a sua recaptura ou substituição até a localização precisa ser alcançada^9^.

Muitas vezes, várias molas são necessárias para a oclusão adequada do vaso, a depender do cenário ou do tamanho da lesão^9^.

- Stent

Entre as técnicas de exclusão vascular, além da embolização, há o uso de stents recobertos e não recobertos. Esses stents consistem em uma estrutura metálica com material biocompatível^9^. Os stents utilizados com maior frequência são autoexpansíveis e expansíveis por balão^1^.

O benefício de usar um stent recoberto é que ele fornece um novo lúmen através da artéria esplênica e exclui a lesão vascular^9^. Também minimiza o infarto esplênico e as complicações da possível formação de abscesso com a embolização da mola^1^.

Essa técnica é particularmente útil no contexto de um aneurisma com um pescoço largo. Se o pescoço é grande, o uso de embolização com mola convencional é imprudente, por causa do aumento do risco de a mola se deslocar, o que pode levar potencialmente à embolização de outras estruturas importantes ou à trombose da artéria principal. Uma desvantagem conhecida dos stents recobertos e que pode limitar seu uso é a dificuldade em avançar os stents através de vasos pequenos ou tortuosos. Por isso, muitas vezes são reservados para locais proximais mais acessíveis^19^.

- Mola com stent

Uma combinação de várias técnicas de tratamento pode ser necessária para alguns casos, particularmente para AAEs gigantes ou pacientes com comorbidades^1^, como embolização com mola assistida por stent. Esta é uma combinação de técnicas usada em certas lesões anatomicamente desafiadoras. Combina o uso de stent de metal e molas. O stent de metal é implantado em toda a lesão para servir como um andaime, e um cateter é, então, colocado através da malha do stent. A segurança de embolização com mola melhorou marcadamente porque as molas estão agora “engaioladas” atrás do stent^9^.

- Embolização líquida

A embolização líquida é feita com agentes que se moldam ao saco aneurismático. Utiliza-se um cateter de entrega ou injeção percutânea direta. Os casos em que essa técnica é benéfica incluem lesões com alto fluxo sanguíneo e lesões em que a posição desejada do dispositivo embólico é mais distal à ponta do cateter de introdução^9^.

Agentes formadores de moldes são usados, como o cianoacrilato de n-butila e o etileno copolímero de álcool vinílico, por meio de injeção controlada. O material flui, inicialmente, e endurece dentro do lúmen da lesão vascular. Além disso, agentes como esponjas de gelatina e trombina (trombina JMI; King Pharmaceuticals, Bristol, Tenn) podem ser injetados para iniciar a cascata de coagulação para trombose da lesão^9^.

- Implante de plugue

Outro método para oclusão vascular envolve uma estrutura metálica complexa, como o plugue vascular Amplatzer (St Jude Medical). Consiste de uma rede de nitinol tridimensional que é avançado por meio de um cateter de entrega. Uma vez no local desejado, o plugue é implantado, desparafusando um fio de segurança, destacável. Ele é projetado para implantação precisa com o uso de um único dispositivo de oclusão autoexpansível. Agentes líquidos injetáveis ​​também podem ser usados conjuntamente^9^.

### Acompanhamento pós-embolização

O acompanhamento de imagem em intervalos de 1 ano foi proposto, devido ao risco de 20% de reperfusão após embolização com mola bem-sucedida. E, se a reperfusão ocorre, o saco aneurismático é novamente exposto a pressões sistêmicas, e pode, mais uma vez, estar em risco de ruptura^9^.

### Complicações da embolização

As complicações mais frequentes da embolização transcateter são migração da mola, ruptura do aneurisma, infarto intestinal, febre, infarto esplênico e abscesso^1^.

A maioria dos procedimentos endovasculares (80% a 90%) é tecnicamente bem-sucedida, com apenas um pequeno grau de infarto esplênico. O fluxo colateral, predominantemente através das artérias gástricas curtas, mantém a perfusão. Entretanto, os riscos de infarto esplênico aumentam com embolizações mais distais^9^.

A pancreatite isquêmica é outra complicação potencial, decorrente da oclusão de ramos da artéria pancreática, mas raramente ocorre se houver suprimento arterial colateral intacto^9^.

Outra complicação teórica associada com o tratamento de AAE é a síndrome da sepse pneumocócica. No momento, não há recomendação publicada sobre o uso da vacinação profilática contra organismos encapsulados no contexto de embolização da artéria esplênica^9^.

Além disso, um aneurisma pode se recanalizar, apesar de a embolização ser bem-sucedida. Nesses casos, pode ser preferível a reembolização ou o tratamento cirúrgico abdominal aberto^1^. Há até um relato de caso de migração da mola para o estômago^46^.

### Síndrome pós-embolização

A síndrome pós-embolização (SPE) pode se manifestar como febre, dor abdominal^25^, íleo, disfunção plaquetária, contagem elevada de leucócitos^47^, derrame pleural e, possivelmente, pancreatite após o infarto do baço, e é a complicação mais comum após correção endovascular. Pode, potencialmente, requerer hospitalização prolongada. Sintomas da SPE são relatados em até 30% dos pacientes^25^. Porém, não se correlaciona com o infarto esplênico. Alguns pacientes completamente assintomáticos podem ter evidência de infarto esplênico em exames rotineiros de imagem pós-procedimento^7^.

Pacientes com AAEs distais parecem estar em maior risco para SPE e/ou infarto esplênico assintomático. A vigilância pós-procedimento para infarto esplênico e formação de abscessos é justificada. Pacientes com SPE têm redução dos sintomas com o tempo, e a intervenção cirúrgica não é necessária^7^.

## CONSIDERAÇÕES FINAIS

Os AAEs são uma patologia rara, mas potencialmente fatal, com mortalidade alta, principalmente em pacientes grávidas, após a ruptura. Seu diagnóstico costuma ser tardio e necessita de uma alta taxa de suspeição. A preferência atual é pelo tratamento endovascular (exceto quando em choque hemorrágico, caso em que, por enquanto, costuma-se optar pela laparotomia), com baixa mortalidade e morbidade no curto prazo, realizado sob anestesia local e que permite rápida recuperação após um curto período de internação hospitalar. Ainda não se sabe qual a melhor técnica endovascular, seja por embolização com ou sem stent, exclusão com stent recoberto, injeção de trombina, injeção de Gelfoam, colocação de cola, colocação de plugue, injeção de partícula ou injeção de álcool polivinílico, e necessita-se de estudos prospectivos maiores para se saber qual técnica é a mais eficaz e segura.
